# Non-Specific Root Transport of Nutrient Gives Access to an Early Nutritional Indicator: The Case of Sulfate and Molybdate

**DOI:** 10.1371/journal.pone.0166910

**Published:** 2016-11-21

**Authors:** Anne Maillard, Elise Sorin, Philippe Etienne, Sylvain Diquélou, Anna Koprivova, Stanislav Kopriva, Mustapha Arkoun, Karine Gallardo, Marie Turner, Florence Cruz, Jean-Claude Yvin, Alain Ourry

**Affiliations:** 1 Normandie Université, Caen, France; 2 UNICAEN, UMR 950 Ecophysiologie Végétale, Agronomie et nutritions N, C, S, Esplanade de la Paix, Caen, France; 3 INRA, UMR 950 Ecophysiologie Végétale, Agronomie et nutritions N, C, S, Esplanade de la Paix, Caen, France; 4 University of Cologne, Botanical Institute and Cluster of Excellence on Plant Sciences (CEPLAS), Cologne, Germany; 5 Centre Mondial d’Innovation, CMI, Groupe Roullier, Saint-Malo, France; 6 INRA, UMR1347 Agroécologie, Pôle GEAPSI, Dijon, France; 7 VEGENOV-BBV, Saint Pol de Léon, France; United States Department of Agriculture, Agricultural Research Service, UNITED STATES

## Abstract

Under sulfur (S) deficiency, crosstalk between nutrients induced accumulation of other nutrients, particularly molybdenum (Mo). This disturbed balanced between S and Mo could provide a way to detect S deficiency and therefore avoid losses in yield and seed quality in cultivated species. Under hydroponic conditions, S deprivation was applied to *Brassica napu*s to determine the precise kinetics of S and Mo uptake and whether sulfate transporters were involved in Mo uptake. Leaf contents of S and Mo were also quantified in a field-grown S deficient oilseed rape crop with different S and N fertilization applications to evaluate the [Mo]:[S] ratio, as an indicator of S nutrition. To test genericity of this indicator, the [Mo]:[S] ratio was also assessed with other cultivated species under different controlled conditions. During S deprivation, Mo uptake was strongly increased in *B*. *napus*. This accumulation was not a result of the induction of the molybdate transporters, Mot1 and Asy, but could be a direct consequence of *Sultr1*.*1* and *Sultr1*.*2* inductions. However, analysis of single mutants of these transporters in *Arabidopsis thaliana* suggested that other sulfate deficiency responsive transporters may be involved. Under field conditions, Mo content was also increased in leaves by a reduction in S fertilization. The [Mo]:[S] ratio significantly discriminated between the plots with different rates of S fertilization. Threshold values were estimated for the hierarchical clustering of commercial crops according to S status. The use of the [Mo]:[S] ratio was also reliable to detect S deficiency for other cultivated species under controlled conditions. The analysis of the leaf [Mo]:[S] ratio seems to be a practical indicator to detect early S deficiency under field conditions and thus improve S fertilization management.

## Introduction

It is usually assumed that plants need to maintain a certain level of homeostasis between mineral nutrients. As a consequence, plants must constantly acquire nutrients from soil solution by a complex network of root specific transporters whose expression is usually up-regulated during deficiency (See [1] for N, [2] for K, [3] for S). However, some nutrients might compete for the active site of non-specific transporters [4]. For example, non-selective cation uptake systems that are able to transport both Na^+^ and K^+^ have been identified in plant root cells, such as HKT1 (a high affinity K^+^ transporter), LCT1 (a low-affinity cation transporter) or NSC (non-selective cation channels) [5]. As a consequence, at higher levels of Na^+^, plants will take up Na^+^ instead of K^+^ [4]. Another case of interactions between competitive ions present in the rhizosphere concerns sulfate (SO_4_^2-^), molybdate (MoO_4_^2-^), selenate (SeO_4_^2-^) and tungstate (WO_4_^2-^) [6, 7]. Molybdate uptake has long been assumed to occur through the sulfate transporters [7, 8]. Indeed, these two anions are chemical analogs and both possess a double negative charge, a tetrahedral structure, a similar size, and hydrogen-bonding properties [9, 10] and therefore may compete for the binding site of the same transporters [11].

The sulfate transporter family consists of 12 genes in *Arabidopsis* that can be subdivided into four groups according to sequence similarities (for review see [3, 12]). The members of the first group, showing a high-affinity for sulfate, are assumed to be principally responsible for primary root uptake. The members of the second group are localized in the root and shoot tissues and are considered as low-affinity sulfate transporters responsible for translocation of sulfate between roots and shoots. The transporters of the third group are localized in plastid envelopes and seem to be responsible for the import of sulfate into the organelles, but have also other specialized functions, e.g. during *Rhizobia* symbiosis in *Lotus japonicus* [13]. Transporters of group four are localized in the tonoplast and are assumed to be responsible for the efflux of sulfate from the vacuoles into the cytoplasm [12]. According to Kataoka *et al*. [14], *AtSultr4*.*1* plays a major role in vacuolar efflux of sulphate while *AtSultr4*.*2* could have a supplementary function. A fifth group comprising two isoforms in *Arabidopsis* (*AtSultr5*.*1* and *AtSultr5*.*2*) has been initially annotated as sulfate transporters but their sequences are considerably shorter, around 450 amino acids, compared to nearly 650 amino acids for the other groups [3] and they do not include the sulfate transporter anti-sigma domain (STAS), which is critical in the transport of sulfate and the stability of transporters [15]. Thus, this latest group is different enough to be considered as an independent family (less than 13% identity with other groups) [15], and indeed, another function has been proposed (see below). Most sulfate transporters (for example *Sultr1*.*1*, *Sultr1*.*2*, *Sultr2*.*1*, *Sultr4*.*1* and *Sultr4*.*2*) are strongly up-regulated by S deficiency through increased gene expression as demonstrated in numerous plant species [6, 16, 17, 18, 19]. Fitzpatrick *et al*. [20] found that uptake of molybdate in *Stylosanthes hamata* can be achieved by SHST1, a high–affinity sulfate transporter from group 1. Molybdate uptake was not reduced when challenged with a competitive anion such as sulfate, whereas the sulfate uptake *via* SHST1 was reduced in presence of molybdate [20]. The transporter from group 5, previously annotated SULTR5.2, was then identified as the first molybdate transporter in *Chlamydomonas reinhardtii* [21] and *Arabidopsis* [10, 22, 23] and was therefore renamed MOT1. However, the localization and the involvement of MOT1 in the uptake of molybdate remain unclear. Indeed, Tomatsu *et al*. [22], using BY2 cells of *Nicotiana tabacum* L. expressing MOT1 fused to GFP under control of the cauliflower mosaic virus 35S RNA promoter, suggested the localization of MOT1 in plasma membranes and endomembrane presumably in the secretory and/or endocytic pathways. Baxter *et al*. [23] established its localization in the mitochondria from the analysis of N-terminal mitochondrial targeting sequence further confirmed by the expression of MOT1 tagged with GFP in protoplasts and transgenic *Arabidospis*. A second molybdate transporter, MOT2 (previously named SULTR5.1) was then described in *Arabidopsis* as being involved in molybdate export from vacuole to cytosol, especially during translocation of molybdate into maturing seeds [10, 24]. Another molybdate transporter also named CrMOT2 has been described in *Chlamydomonas reinhardtii* [25] but it is different from its namesake described previously. The knockdown expression of this *CrMot2* gene leads to a reduction of molybdate uptake proportional to the decrease in its transcript level [25]. More recently, a homolog of *CrMot2*, (named *Asy* for Abnormal Shoot in Youth) has been reported in higher plants [26]. Its localization in the plasma membrane together with the severe phenotypes in *asy* mutants supplied with sufficient Mo suggest that the ASY transporter is crucial for molybdate uptake [26].

Balik *et al*. [27] have demonstrated an antagonistic relationship between the sulfate and molybdate uptake by plants. Previous studies in *Brassica napus* [27] and *Brassica juncea* [7] showed an accumulation of Mo in response to S limitation. These authors suggested that molybdate binds to the active sites of the root sulfate transporters, which was favoured by the lack of competition with sulfate. Moreover, Shinmachi *et al*. [6] showed under field conditions that a significant accumulation of Mo in *Triticum aestivum* plants with low S fertilization was concomitant with the increased expression of root sulfate transporters. It has also been shown that mineral S application leads to a significant decrease in Mo concentration and uptake in *B*. *juncea* [7, 28] and *Brassica oleracea* [29] or that the absence of sulfate in nutrient medium causes a significant accumulation of Mo in *Solanum lycopersicum* [11]. Considering the effects of S deficiency on the Mo accumulation previously described, it could be hypothesized that the leaf [Mo]:[S] ratio might serve as an indicator of S nutrition to predict S deficiency. For S-demanding crops such as oilseed rape, S deficiency provokes multiple plant physiological changes leading to losses in yield and seed quality through modified lipid and protein composition of seeds [30, 31, 32, 33, 34]. It has already been suggested that the analysis of plants rather than soil via total S, SO_4_^2-^ or N:S, malate:sulfate or ([Cl^-^]+[NO_3_^-^]+[PO_4_^3-^]):[SO_4_^2-^] ratios could be used to detect plant S requirements (for review see [35, 36] but these parameters are affected by growth stage and growth rates or need laboratory analyses, making them unreliable as diagnostic indicators [35, 37]. It is therefore essential to find an accurate indicator of plant S status that is suitable as a reliable and relevant diagnostic tool under field conditions in order to evaluate the S requirements of *B*. *napus* and other important cultivated species in the context of the high risk of S deficiency.

The main objectives of this work were to study the interaction effects between S and Mo uptake and accumulation in oilseed rape. The first objective was to confirm that Mo accumulation occurred during S deprivation in oilseed rape under controlled conditions, with a precise kinetic description of Mo uptake together with the transcript levels of genes encoding sulfate and molybdate transporters. Additionally, the putative molybdate uptake by sulfate transporters was assessed in *atsultr1;1and atsultr1;2 Arabidopsis* mutants grown under S deficiency. The second objective was then to investigate how the [Mo]:[S] ratio in leaves of oilseed rape grown under field conditions was affected by S and N fertilization, and consequently if it constitutes a relevant early indicator to predict the risk of S deficiency at a larger scale. Finally, the last objective was to test the functionality of the [Mo]:[S] ratio as an indicator of S deficiency in other cultivated species such as *B*. *oleracea*, *T*. *aestivum*, *Zea mays*, *Pisum sativum* and *Solanum lycopersicum* grown under controlled conditions.

## Materials and Methods

### Hydroponic experiments and tissue sampling

Seeds of *B*. *napus* L. var. Boheme were germinated on perlite over demineralized water for seven days in the dark and then five days under natural light. Just after the first leaf emergence, seedlings were transferred to hydroponic conditions (18 seedlings per 20 L-plastic tank) in a greenhouse, between October and December, with a thermoperiod of 20°C (day) and 15°C (night). Natural light was supplemented with high-pressure sodium lamps (Master Greenpower T400W, Philips, Amsterdam, Netherlands) (350 μmol m^-2^ s^-1^ of photosynthetically active radiation at the canopy height) for 16 h. The aerated nutrient solution contained: 3.75 mM KNO_3_, 0.5 mM MgSO_4_, 0.5 mM CaCl_2_, 0.25 mM KH_2_PO_4_, 0.2 mM EDTA-2NaFe, 14 μM H_3_BO_3_, 5 μM MnSO_4_, 3 μM ZnSO_4_, 0.7 μM CuSO_4_, 0.7 μM (NH_4_)_6_Mo_7_O_24_, 0.1 μM CoCl_2_, 0.04 μM NiCl_2_ and buffered to pH 6.6 with 0.91 mM CaCO_3_. This solution was renewed according to the rate of NO_3_^-^ depletion monitored daily by using NO_3_^-^ test strips (Merck Millipore, Darmstadt, Germany) in order to maintain optimal nutrition conditions.

After 4 weeks of growth, plants were separated into two batches supplied with a modified nutrient solution chosen in order to achieve S deficiency and to maintain the same concentration of other nutrients: (i) control plants (+S) were grown with 508.7 μM SO_4_^2-^, (ii) S-deprived plants (-S) were grown with 8.7 μM SO_4_^2-^ (S1 Table). These nutrient solutions were also renewed according to NO_3_^-^ depletion by monitoring the NO_3_^-^ level in the tank (at the end of the experiment this was about every two days).

Four independent samples each consisting of four individual plants were harvested at day 0 and after 1, 2, 3, 7, 13, 21 and 28 days of treatment. Leaves present at the beginning of treatment applications (d0) were identified and marked, and these organs were referred to as “emerged leaves”, while leaves appearing during treatments were harvested separately as “new emerging leaves”. At each date of harvest, whole roots from control and depleted plants (-S) were also harvested. Thereafter, leaves, petioles and roots were frozen in liquid nitrogen and stored at -80°C for further analysis. It must be pointed out that roots were freeze dried without any prior rinsing and therefore the root analyses include whatever is bound outside the tissue as well as internal concentration of elements. An aliquot of each tissue was freeze-dried for dry weight (DW) determination and ground, using an oscillating grinder (MM400, Retsch, Haan, Germany), to fine powder for element analysis.

*Arabidopsis* plants (Col-0 as wild type, SALK_093256 as *sultr1*.*1* and *sel1-8* [38] as *sultr1*.*2*) were grown for 17 days weeks on vertical agarose plates with modified Long Ashton nutrient solution with full (0.75 mM) or limited (0.075 mM) sulfate in controlled environment room under a long day 16-h-light/8-h-dark cycle at constant temperature of 22°C, 60% relative humidity, and light intensity of 120 μE s^–1^m^–2^. The sel1-8 mutant has been isolated from a genetic screen on selenate tolerance using an EMS mutagenized Col-0 seeds and contains a point mutation in the coding sequence [38]

### Field experiments and plant sampling

#### Study of different fertilization rates on an S deficient field

The experimental site was selected from a previous study [39] showing an S deficiency in *B*. *napus* L. grown in 2009. This field of 11.3 ha was located at Ondefontaine, France (48°59'18.69" N, 00°41'56.70" W). A winter oilseed rape crop (*B*. *napus* L., 95% cv DK Exstorm and 5% cv Alicia) was sown with a density of 35–40 plants m^-2^ on 27 august 2013. The previous crop was spring barley (*Hordeum vulgare* L.). Chemical analyses of the loam soil provided an S content of 220 mg kg^-1^ and a pH value of 6.4. Before crop establishment, organic fertilizer (40 m^3^ ha^-1^ of bovine manure) was applied to the whole field corresponding to 122 kg N ha^-1^ and 44 kg S ha^-1^. The field was separated into seven randomized plots fertilized with different doses of N and S mineral fertilizer. One plot was unfertilized. Other plots were fertilized on 27 February 2014 with three different doses of S fertilizer at 0, 12 and 36 kg S ha^-1^ with a combined fertilizer comprising ammonium and nitrate and sulfuric anhydride, 26% N and 36% SO_3_ and complemented with ammonium nitrate to obtain 65 kg N ha^-1^ on each plot. Then, on 27 March 2014, a second N fertilization with 60 kg N ha^-1^ (liquid N containing 50% urea, 25% ammonium and 25% nitrate) was applied to three plots, which thus received 125 kg N ha^-1^ after two fertilizer applications.

Five harvests were performed between January and July 2014: before fertilization (at the rosette stage and at the start of stem elongation (GS30), January 30^th^ 2014 and February 12^th^ 2014), 15 days after S fertilization (start of the visible bud stage (GS50), March 14^th^ 2014), 47 days after S fertilization (silique formation stage (GS70), April 15^th^ 2014) and finally after 60 days (silique formation stage (GS70), April 28^th^ 2014). Two batches of leaves comprising mature leaves (from the lower canopy) and young leaves (from the higher canopy), each of them consisting of three replicates of 20 leaves, were randomly collected from each plot. Leaves were freeze-dried for DW determination and ground, using an oscillating grinder (MM400, Retsch, Haan, Germany) to fine powder for further analysis.

#### Study of 45 commercial crops before fertilization and flowering

Forty-five commercial crops of oilseed rape were selected in France according to different locations (given in S1 Fig), different agricultural practices (dose of fertilizers, previous crop, tillage), and under contrasting soil and climate conditions that may affect SO_4_^2-^ leaching and hence SO_4_^2-^ availability. The farmers, identified with the help of DATAGRI (Lyon, France), collaborated in the present study. Crops have been numbered arbitrarily, from 1’ to 45’. Two batches of leaves comprising mature leaves (from the lower canopy) and young leaves (from the higher canopy), each of them consisting of 20 leaves, were randomly collected from each of the 45 fields in February 2014, just at the end of winter corresponding to the start of stem elongation (GS30) and before fertilization. Leaves were freeze-dried before laboratory processing.

### Multispecies experiment under controlled conditions

In order to assess whether data obtained with *B*. *napus* could be extrapolated to other species, experiments using *B*. *oleracea*, *T*. *aestivum*, *Z*. *mays*, *S*. *lycopersicum* and *P*. *sativum* grown under controlled conditions were conducted under different culture conditions described in S1 File with optimal and sub-optimal S alimentation. Leaves were harvested and frozen immediately in liquid nitrogen and stored at -80°C until freeze-drying for further analysis.

### Element analysis by mass spectrometry

For the analysis of total N and S contents, an aliquot of around 4 mg DW of each plant organ sample was placed in tin capsules for total N and S analysis in order to analyze between 60 and 80 μg N. The total N amounts and S amounts in plant samples were determined with a continuous flow isotope mass spectrometer (Nu Instruments, Wrexham, United Kingdom) linked to a C/N/S analyzer (EA3000, Euro Vector, Milan, Italy). The total N or S amount (N_tot_ or S_tot_) in a tissue “i” at a given time “t” was calculated as:

$$N_{tot}\;\left(or\;S_{tot}\right)=\frac{\%\;N_{i,t}\left(or\;S_{i,t}\right)\times DW_{i,t}}{100}$$

B, Na, Mg, P, S, K, Ca, Mn, Fe, Ni, Cu, Zn and Mo in samples were quantified by High Resolution Inductively Coupled Plasma Mass Spectrometry (HR ICP-MS, Thermo Scientific, Element 2^™^, Bremen, Germany) with prior microwave acid sample digestion (Multiwave ECO, Anton Paar, les Ulis, France) using 800 μL of concentrated HNO_3_ (Thermo Fischer, Illkirch, France), 200 μL of H_2_O_2_ (SCP SCIENCE, Quebec, Canada) and 1mL of Milli-Q water for 40 mg DW, except for *Arabidopsis* for which only 20 mg DW were used. For the determination by HR ICP-MS, all the samples were spiked with two internal-standard solutions, gallium and rhodium (SCP SCIENCE, Quebec, Canada), for a final concentration of 10 and 2 μg.L^-1^, respectively, diluted to 50 ml with Milli-Q water to obtain solutions containing 2.0% (v/v) of nitric acid, then filtered on a 40 μm teflon filtration system (Courtage Analyses Services, Mont-Saint-Aignan, France). Quantification of each element was performed using external standard calibration curves. The quality of mineralization and analysis were checked using a certified reference material of *Citrus* leaves (CRM NCS ZC73018, Sylab, Metz, France). The amount of each element in each tissue was then calculated as previously explained for N and S.

### Reverse transcription (RT) and q-PCR analysis

Total RNA was extracted from 200 mg of root and leaf fresh matter as previously described [36]. For RT, 1 μg of total RNA was converted to cDNA with an iScript cDNA synthesis kit according to the manufacturer’s protocol (Bio-Rad, Marne-la-Coquette, France). Q-PCR amplifications were performed using specific primers (Table 1) for each housekeeping gene (*EF1-α* and *18S rRNA*) and target genes (*BnaSultr1*.*1*, *BnaSultr1*.*2*, *BnaASY*, *BnaMot1*). Q-PCRs were performed with 4 μl of 100x diluted cDNA, 500 nM of primers, and 1x SYBR Green PCR Master Mix (Bio–Rad, Marne–la–Coquette, France) in a real-time thermocycler (CFX96 Real Time System, Bio–Rad, Marne–la–Coquette, France). A program of three steps composed of an activation step at 95°C for 3 min and a denaturing step of 40 cycles at 95°C for 15 s followed by an annealing and an extending step at 60°C for 40 s was used for all pairs of primers (Table 1). For each pair of primers, a threshold value and PCR efficiency was determined using a cDNA preparation diluted >10-fold. For all pairs of primers, PCR efficiency was around 100%. The specificity of PCR amplification was examined by monitoring the presence of the single peak in the melting curves after q-PCRs and by sequencing the q-PCR product to confirm that the correct amplicon was produced from each pair of primers (Eurofins, Ebersberg, Germany). For each sample, the subsequent q-PCRs were performed in triplicate. The relative expression of the genes in each sample was compared with the control sample (corresponding to control plants at d0) and was determined with the delta delta Ct (ΔΔCt) method using the following equation: relative expression = 2^-ΔΔCt^, with ΔΔCt = ΔCt_sample_-ΔCt_control_ and with ΔCt = Ct_target gene_−Ct_housekeeping gene_ (for calculations, we considered the geometric mean between Ct of the housekeeping genes), where Ct refers to the threshold cycle determined for each gene in the exponential phase of PCR amplification. Using this analysis method, relative expression of the target gene in the control sample was equal to 1 [40], and the relative expression of other treatments was then compared with the control.

**Table 1:** Accession number and Q-PCR primer sets. *EF1-α* and *18S rRNA* were housekeeping genes used for relative gene expression by Q-PCR analysis.

Gene	Accession number	Forward	Reverse
*EF1-α*	DQ312264	5’-gcctggtatggttgtgacct-3’	5’-gaagttagcagcacccttgg-3’
*18S rRNA*	X16077	5’-cggataaccgtagtaattctag-3’	5’-gtactcattccaattaccagac-3’
*BnaSultr1.1*	AJ416460	5′-agatattgcgatcggaccag-3′	5′- gaaaacgccagcaaagaaag-3′
*BnaSultr1.2*	AJ311388	5′-ggtgtagtcgctggaatggt-3′	5′-aacggagtgaggaagagcaa-3′
*BnaAsy*	XM009145653	5′-tcacgctcatctcccaaagt -3′	5′-agtcctacacacgcctcaaa -3′
*BnaMot1*	NM128127	5′-ctcgccaggatttggactta-3′	5′-agatccccaacacgaacaag-3′
*AtSULTR1.1*	At4g08620	5’-cggccatctacttttccaac-3’	5’-gcattttcttgctcctctcg-3’
*AtSULTR1.2*	At1g78000	5’-atccgttttcaaagcagctc-3’	5’-tcaagaatgatgcaccaatga-3’
*AtSDI1*	At5g48850	5’-tccctgtggagacactcctt-3’	5’-ccatctccgggttcttctct-3’
*AtAPR3*	At4g21990	5’-ccaatcaagtatccatcagagaag-3’	5’-ccgaacaagattcaagaaagatg-3’
*AtUBQ10*	At4g05320	5’-ctgcgactcagggaatcttc-3’	5’-ttgtgccattgaattgaacc-3’

Expression analysis of *Arabidopsis* was performed as described previously [41] with RNA isolated from root material and using ubiquitin *UBQ10* for normalization (see Table 1 for primer sequences).

### Statistical analysis

For each measurement, hydroponic experiments on *B*. *napus* were conducted with four independent biological replicates constituted of four individual plants, except for q-PCR analysis, which was conducted with three biological replicates. Data are given as mean ± SE for n = 4 (or n = 3 for q-PCR data). All data of the hydroponic experiment were analyzed by Student’s test (Excel software) and marked by one or several asterisks when significantly different between controls and S-deprived plants (*P<0.05, **P<0.01, ***P<0.001). Under field conditions, for the study of different fertilization rates on an S deficient field, three replicates corresponding to a pool of 20 leaves of 20 independent plants were collected. Data are given as mean ± SE for n = 3 and were analyzed by Student’s test (Excel software) and marked by different letters when they were significantly different between treatments at a given date, at *P*<0.05. All experiments on different species in controlled conditions were conducted with four independent biological replicates, except for *P*. *sativum* culture with three replicates. Data are reported as mean ± SE for n = 4 (or n = 3 for *P*. *sativum* data). All data of the multispecies experiment were analyzed by Student’s test (Excel software) and marked by one or several asterisks when significantly different between controls and S-deprived plants (*P<0.05, **P<0.01, ***P<0.001).

## Results

### S deprivation reduced S, N, K, Ca, B and Na uptake but strongly increased Mo uptake

**Fig 1:**
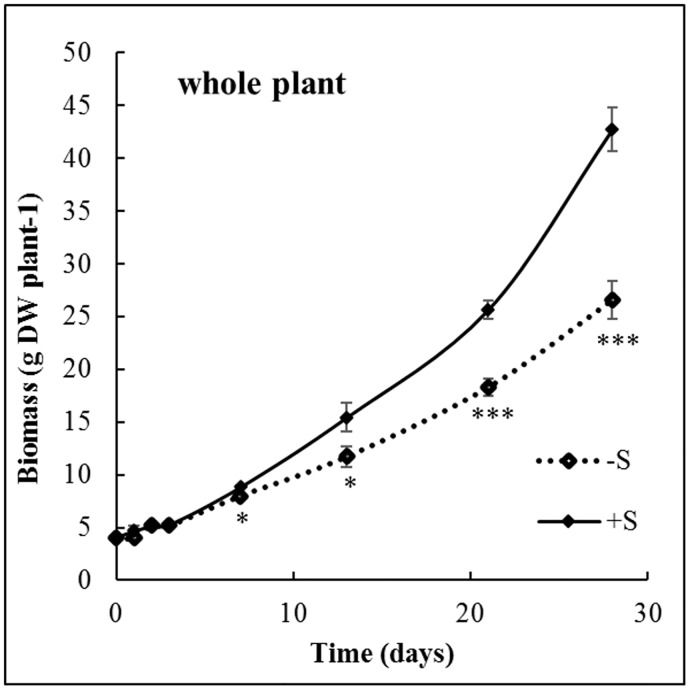
Change in whole plant biomass (g DW plant^-1^) of *B*. *napus* L. in control (+S, black line), and S-deprived (-S, dashed line) plants during the 28 days of the experiment. Data are given as the mean ± s.e. (n = 4). *, ** and *** indicate significant difference between control and S-deprived plants for *P*<0.05, *P*<0.01 and *P*<0.001, respectively.

**Fig 2:**
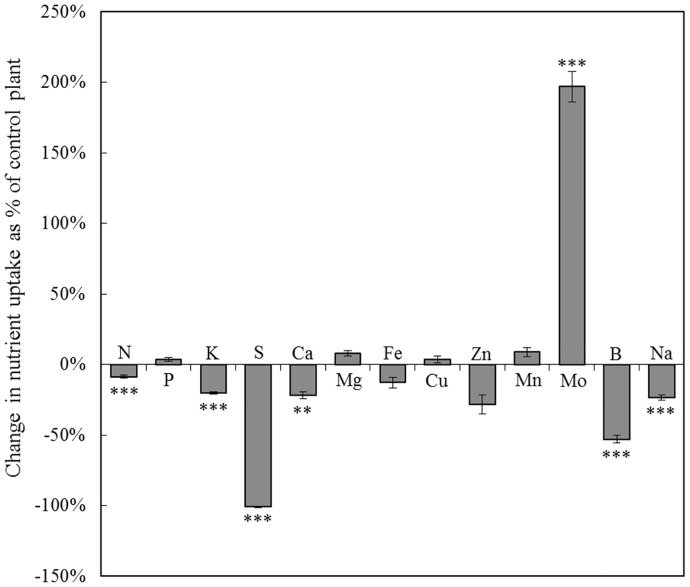
Changes in uptake of macro (N, P, K, S, Ca and Mg) and micronutrients (Fe, Cu, Zn, Mn, Mo, B and Na) in *B*. *napus* after 21 days of S deprivation. A theoretical uptake by S-deprived plants was calculated for each nutrient, which reflects the uptake expected by S-deprived plants if DW was identical to control plants. Change in nutrient uptake was calculated as the difference between S-deprived and control plants and expressed as % of uptake achieved by control plants. Data are given as the mean ± s.e. (n = 16). *, ** and *** indicate significant differences between control and S-deprived plants for *P*<0.05, *P*<0.01 and *P*<0.001, respectively.

*B*. *napus* plant growth rates were similar for control and S-deprived plants until the 7^th^ day of S deprivation (Fig 1) and were then significantly reduced. For example, after 21 days, the biomass of S-deprived plants decreased by 29% compared to the biomass of control plants. To overcome the biomass reduction, a theoretical uptake by S-deprived plants was calculated for each nutrient (Fig 2) using the DW of control plants and the nutrient concentration measured in S-deprived plants. This theoretical uptake reflects the uptake expected by S-deprived plants if DW production was identical to control plants. As expected, after 21 days of S deprivation, no significant of S occurred. The uptake of some nutrients was reduced in line with the reduction in the growth rate or more strongly: for N (-8.7 ± 1.06%), K (-20.2 ± 0.88%), Ca (-22.0 ± 2.41%), Na (-23.4 ± 1.85%) and B (-52.9 ± 2.82%). Conversely, S deprivation strongly increased the uptake of Mo by 197.0 ± 10.73%. The uptake of P, Mg, Fe, Cu, Zn and Mn were not significantly affected by S deprivation.

### S deprivation strongly and immediately increased uptake of Mo without induction of molybdate transporter genes

**Fig 3:**
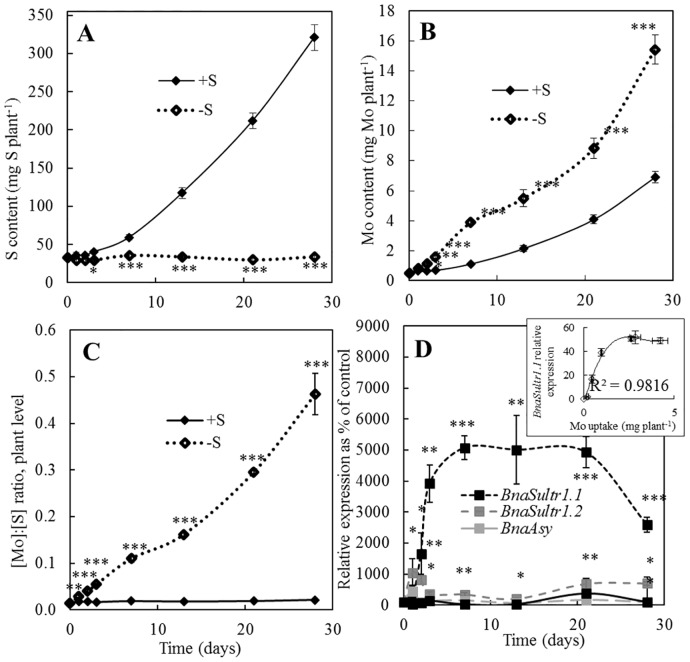
(A) Cumulated S and (B) Mo contents (mg plant^-1^) in *B*. *napus* control (+S, black line) or S-deprived (-S, dashed line) plants. (C) Kinetic of the [Mo]:[S] ratio in *B*. *napus* from control (+S, black line) or S-deprived (-S, dashed line) plants. (D) Effect of S deprivation on relative expression of root transporters: *BnaSultr1*.*1*, *BnaSult2*.*1*, *BnaAsy* and *BnaMot1* genes expressed in % of control plants. The insert provides the correlation between Mo uptake and the relative expression of *BnaSultr1*.*1*. Data are given as the mean ± s.e. (n = 4 or n = 3 for q-PCR data). *, ** and *** indicate significant differences between control and S-deprived plants for *P*<0.05, *P*<0.01 and *P*<0.001, respectively.

In S-deprived *B*. *napus*, plant S content (Fig 3A) remained at a nearly steady state level, revealing a lack of significant S uptake from potential trace amounts in the nutrient solution. In control plants, the plant S content steadily increased with time and was significantly higher than in S-deprived plants from 3 days of S deprivation. Plant Mo content (Fig 3B) increased with time in S-deprived plants, similar to control plants, but at a significantly greater rate; reaching after 28 days 15.43 and 6.93 mg Mo.plant^-1^ in S deprived and control plants, respectively. From the first day, the Mo content was significantly increased by 28% in S-deprived plants relative to control *i*.*e*. before a significant difference in S content that occurred after only 3 days. As a consequence, the [Mo]:[S] ratio at the whole plant level (Fig 3C) significantly increased as early as one day after S deprivation, and was increased from 0.0014 (day 0) to 0.45 after 28 days of S deprivation. In control plants, the [Mo]:[S] ratio remained constant as a function of time.

The relative expression of genes encoding different molybdate (*BnaMot1* and *BnaAsy*) and sulfate (*BnaSultr1*.*1*and *BnaSultr1*.*2*) transporters was quantified in roots during the 28 days of treatment (Fig 3D). A very strong accumulation of *BnaSultr1*.*1* transcripts occurred during the first 7 days and remained at a steady state level between 7 and 21 days. The *BnaSultr1*.*2* transcript level was also significantly up-regulated, but to a lesser extent than *BnaSultr1*.*1* in response to S deprivation from the first day of treatment. In contrast, expression of genes encoding root molybdate transporters (BnaMOT1 and BnaASY) (Fig 3D) was not affected by S deprivation despite the strong increase in Mo uptake by S-deprived plants (Fig 3B). The relative expression of *BnaSultr1*.*1* appeared to be correlated with Mo uptake (polynomial order 2, R² = 0.98) during the 21 days of S deprivation (insert Fig 3D).

To test whether the increase in [Mo] is indeed caused by increased activity of sulfate transporters, we analysed *Arabidopsis thaliana* mutants in *Sultr1*.*1* and *Sultr1*.*2*. The wild type *Arabidopsis* (Col-0), when submitted to S deficiency strongly increased the transcript levels of *AtSultr1*.*1* and *Atsultr1*.*2* as well as those of marker genes for sulfate deficiency *AtSDI1* and *AtAPR3*, which encode for a sulfur responsive gene and an adenosine 5′-phosphosulfate reductase, respectively (Table 2). At the same time, leaf S content was reduced by 55% and leaf Mo content was increased by 571%. In control conditions the *sultr1*.*1* mutant shows leaf S and Mo contents identical to wild type. In S deficient conditions, S (-56%) is decreased to the same degree as in Col-0. The increase in Mo (+433%) leads to similar Mo concentration as in wild type (Table 2). Interestingly, *AtSultr1*.*2* was not up-regulated by S deficiency in this mutant despite similar S concentrations. In the *sultr1*.*2* mutant (Table 2), leaf S content was lower than in Columbia ecotype under ample S supply and was further decreased by S deficiency (-27%), but only to levels found in the wild type. Molybdenum was lower compared to wild type in control conditions, similar to S, and accumulated (+729%) under S deficiency but reached a lower concentration than in wild type or in *sultr1*.*1* KO mutant. In the *sultr1*.*2* mutant the transcript levels of the four genes, *AtSultr1*.*1*, *AtSultr1*.*2*, *AtSDI1* and *AtAPR3* were higher than in wild type and *sultr1*.*1*, and were up-regulated by S deficiency (Table 2). Interestingly, the ratio of [Mo] to [S] is identical in all three genotypes under control conditions, despite the differences in S content, but at S limiting conditions the ratio is higher in wild type than in both mutants. This suggests that both transporters contribute to Mo accumulation but are not the only and/or main drivers.

**Table 2:** Effect of S deficiency on relative expression of genes: *AtSultr1*.*1*, *AtSultr2*.*1*, *AtSDI1* and *AtAPR3* in roots of *sultr1*.*1 and sultr1*.*2* mutants *of Arabidopsis thaliana* expressed relatively to wildtype Col-0 grown under sufficient S supply (Control).

	*Arabidopsis* mutants					
	Col-0		*sultr1*.*1 KO*		*sultr1*.*2 (sel1-8)*	
Gene expression	Control	Low S	Control	Low S	Control	Low S
***AtSultr1*.*1***	1.0±0.20a	15.5±4.99b	0c	0c	13.8±0.23b	74.4±9.40d
**AtSultr1.2**	1.0±0.12a	3.2±0.61b	1.0±0.07a	1.8±1.06a	3.4±0.52b	4.4±0.53b
***AtSDI1***	1.0±0.13a	41.9±14.31b	1.5±0.31a	81.6±37.03b	19.0±2.40b	167.2±16.59c
***AtAPR3***	1.0±0.14a	3.6±0.53b	1.1±0.08a	4.3±0.19b	2.7±0.09a	5.0±0.11c
**Leaf concentration**						
**S (mg.g**^**-1**^ **DW)**	11.8±0.44a	5.3±0.06b	12.7±0.27a	5.6±0.03b	6.9±0.09d	5.0±0.11b
**Mo (ug.g**^**-1**^ **DW)**	28.7±1.22a	192.7±10.49b	31.9±0.55a	170.0±10.49b	16.3±0.33c	134.9±5.07d
**[Mo]:[S].10**^**4**^	24.2±0.12a	365±5.7b	25.14±0.73a	304±18.0c	23.7±0.44a	270±7.4c

In the sultr1.2 mutant the transcript was present, because the gene is inactivated by a point mutation in coding sequence [38]. Leaf concentrations of S and Mo and the [Mo] to [S] ratios (i.e. [Mo]:[S] multiplied by 10^4^ for easier reading) are also given for the same plants. Values from the same line (i.e. between genotypes and S treatments for a given transcript or a nutrient concentration) are significantly different at P<0.05 when marked by different letters. Data are given as the mean ± SE (n = 3 for PCR analysis, n = 4 for S and Mo analysis).

### In leaves of S-deprived plants, Mo and S contents changed in opposite ways

**Fig 4:**
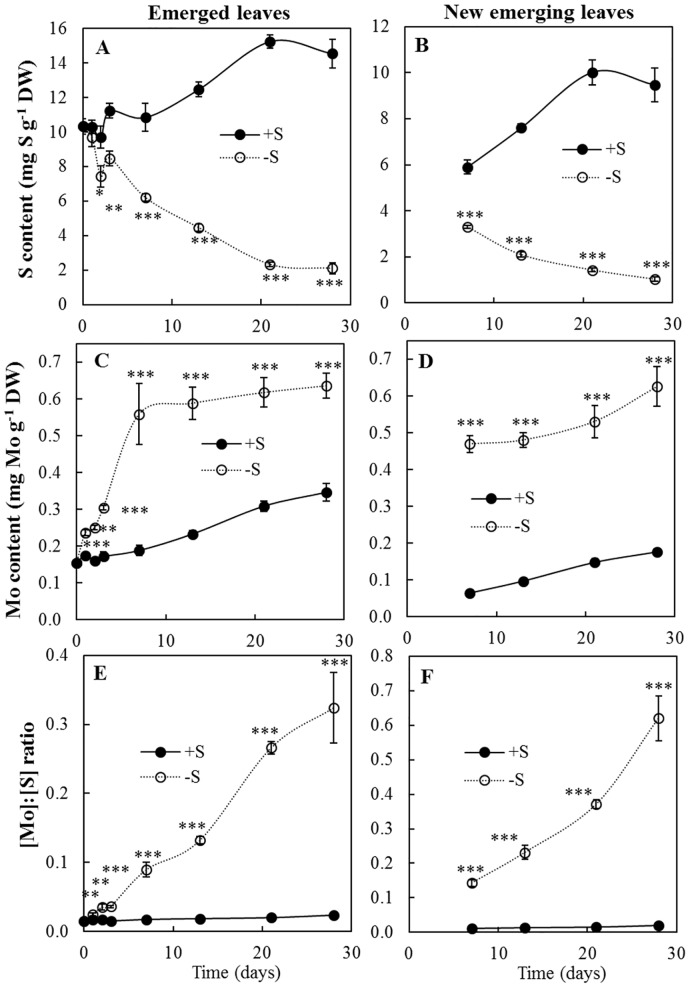
Kinetic of (A-B) S and (C-D) Mo contents (mg g^-1^ DW) and (E-F) the [Mo]:[S] ratio in emerged (on the left) and new emerging (on the right) leaves of *B*. *napus* from control (+S, black line) or S-deprived (-S, dashed line) plants. Data are given as the mean ± s.e. (n = 4). *, ** and *** indicate significant differences between control and S-deprived plants for *P*<0.05, *P*<0.01 and *P*<0.001, respectively.

Under hydroponic conditions, no matter which leaves were assayed (emerged leaves or new emerging leaves), the S content steadily increased with time in control plants (Fig 4A and 4B). Predictably and according to deprivation, in emerged leaves from S-deprived plants the S content significantly decreased as early as 2 days after S deprivation (Fig 4A). The S content decreased with the same pattern in new emerging leaves (Fig 4B). Molybdenum content steadily increased with time in the two groups of leaves of control plants but the accumulation of Mo was significantly and rapidly (from 1 day) increased by S deprivation and until the end of the experiment (Fig 4C and 4D). Compared to control plants, the Mo content in emerged leaves (Fig 4C) was increased by 197% and 84% after 7 and 28 days of S deprivation, respectively. The increase in Mo content was even more pronounced in new emerging leaves (Fig 4D) where it increased by 637% and 253% after 7 and 28 days of S deprivation, respectively. In these two groups of leaves, the [Mo]:[S] ratio (Fig 4E and 4F) strongly increased with time in S-deprived plants and remained at a constant level in control plants. Indeed, there was significant increase of 45% in the [Mo]:[S] ratio in emerged leaves (Fig 4E) after the first day of S deprivation and after 28 days this increase reached 1256%. At the same time, the [Mo]:[S] ratio in new emerging leaves (Fig 4F) was also increased by 3158% in S-deprived plants. The [Mo] and [S] changes on the whole plant level are thus driven by changes in both emerged and new emerging leaves.

### Under field conditions, lower S availability increased leaf Mo content and hence the [Mo]:[S] ratio

**Fig 5:**
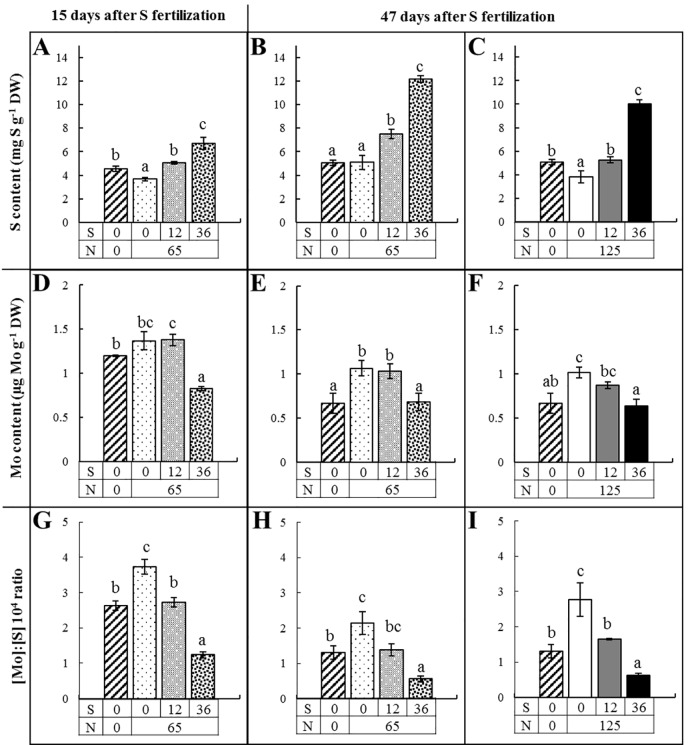
(A, B, C) S content (mg g^-1^ DW), (D, E, F) Mo content (μg g^-1^ DW) and (G, H, I) the [Mo]:[S] 10^4^ ratio (to simplify reading the [Mo]:[S] ratio is presented with a multiplier factor of 10^4^) in mature leaves of *B*. *napus* grown under field conditions after (A, D, G) 15 and (B, C, E, F, H, I) 47 days of fertilization. Plants received no mineral fertilization (hatched bars, 0 kg S.ha^-1^, 0 kg N.ha^-1^), or 0 kg S ha^-1^ (white bar), 12 kg S ha^-1^ (gray bar) or 36 kg S ha^-1^ (black bar) with 65 kg N ha^-1^ (dashed bars) or 125 kg N ha^-1^ (full bars). Within the same graph, letters when different between fertilization treatments indicate significant differences for *P*<0.05.

The S content, Mo content and the [Mo]:[S] ratio were also quantified in mature (Fig 5) and young leaves (S2 Fig) of oilseed rape grown under field conditions, 15, 47 and 60 days after S fertilization. To simplify reading, only the data after 47 days are presented because the results obtained at 60 days showed similar trends. Sulfur content (Fig 5A–5C) was decreased significantly in mature leaves by a reduction in S fertilization (from 36, 12 to 0 kg S ha^-1^), whatever the level of N fertilization (65 or 125 kg N ha^-1^). The highest Mo content (Fig 5D and 5F) was found in mature leaves of plants receiving N fertilization and with no or low S fertilization (0 or 12 kg S ha^-1^). The [Mo]:[S] ratio (Fig 5G–5I) differentiated the plots according to S fertilization: 0, 12 and 36 kg S ha^-1^. Indeed, mature leaves of plants with N fertilization (65 or 125 kg N ha^-1^) and without S had the highest [Mo]:[S] ratio, whereas mature leaves of plants with 36 kg S ha^-1^ had the lowest [Mo]:[S] ratio and mature leaves of plants with 12 kg S ha^-1^ had an intermediate [Mo]:[S] ratio. The same trends were also found in young leaves (S2 Fig) but the effect of S fertilization on the [Mo]:[S] ratio was slightly higher in mature leaves.

Interactions between N and S fertilization were also found. It can be assumed that the growth rate was mostly dependent on the N fertilization rate. For example, 15 days after S fertilization, the biomass of mature leaves was 5.03 ± 0.38 g DW leaf^-1^ in unfertilized plot (N0S0) whereas in plot with 65 kg N ha^-1^ and 0 kg S ha^-1^ (N65S0) the biomass was 6.70 ± 0.01 g DW leaf^-1^ (*P<*0.01). The supply of N mineral fertilization had a direct negative consequence on S content in leaves as a result of the increased growth rate. Indeed, without S supply, N fertilization significantly reduced the leaf S content after 15 days (Fig 5A, N0S0 versus N65S0) or after 47 days (Fig 5C, N0S0 versus N125S0). Consequently, the Mo content in leaves was increased by N fertilization of 65 (Fig 5E, N0S0 versus N65S0 or N65S12) or 125 kg.N ha^-1^ (Fig 5F, N0S0 versus N125S0). As a result, the [Mo]:[S] ratio was significantly lower (Fig 5G, 5H or 5I) in the absence of N mineral fertilization in mature leaves (N0S0), than in N fertilized plots with no S (N65S0 in Fig 5H and N125S0 in Fig 5I), therefore reflecting lower S requirements of plants without N fertilization.

In the same way, plant growth was stimulated by a second N fertilization of 60 kg N ha^-1^ (corresponding to plots with 125 kg N ha^-1^) and the relative S content in mature leaves was reduced. For example, when comparing Fig 5B and 5C, the S content in mature leaves of plants with 125 kg N ha^-1^ was significantly lower (*P*<0.05) than for plants with 65 kg N ha^-1^ and for a given level of S fertilization (0, 12 or 36 kg S ha^-1^). In contrast, the N fertilization rate had no significant effect on the Mo content of mature leaves (Fig 5E compared to Fig 5F). As a result, when comparing mature leaves of plants receiving 125 kg N ha^-1^ (Fig 5I) to 65 kg N ha^-1^ (Fig 5H), the [Mo]:[S] ratio was higher in leaves of plants with a higher N fertilization, regardless of the rate of S fertilization, which also reflected the higher requirement for S of N fertilized plants.

**Fig 6:**
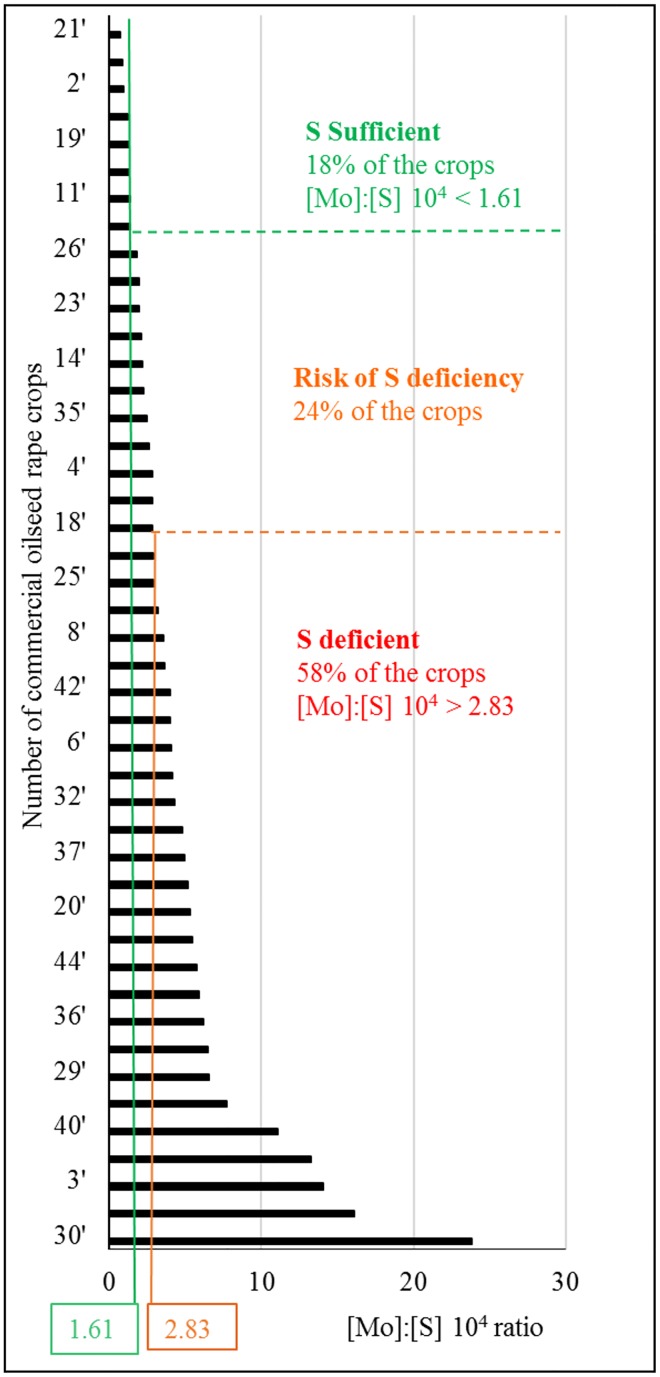
Classification of 45 commercial oilseed rape crops from different locations in France (see S1 Fig) according to their [Mo]:[S] 10^4^ ratio in mature leaves quantified before flowering and S fertilization. Threshold values of the [Mo]:[S] 10^4^ ratio classified these oilseed rape crops into three S status groups: S deficient, at risk of S deficiency and S sufficient plants.

Threshold values of the [Mo]:[S] ratio were then determined 15 days after S fertilization, corresponding to development stage in mature leaves of oilseed rape before flowering, to conclude whether or not S fertilization was required. Threshold values of the [Mo]:[S] ratio were adjusted by factor 10^4^ and calculated as the sum or difference between the means of data presented in Fig 5G and the 95% confidence intervals for S sufficient plots (36 kg S ha^-1^, 1.63) and of S deficient plots (0 kg S ha^-1^, 2.83), respectively. Such thresholds would allow clustering of the plots into three groups: S sufficient (ratio <1.61), at risk of S deficiency (ratio between 1.61 and 2.83) and S deficient (ratio >2.83). This classification into three groups has been tested on 45 commercial oilseed rape crops from different locations in France and for which mature leaves were harvested before flowering and S fertilization (Fig 6). The use of these threshold values suggested that 18, 24 and 58% of the crops were classified as S sufficient, at risk of S deficiency and S deficient, respectively. Because it was previously found that S deprivation strongly decreased N, K, Ca, B and Na uptake under hydroponic conditions (Fig 2), a principal component analysis was performed using the mineral composition of old leaves of theses 45 commercial oilseed rape crops (S3 Fig). While an inverse relationship between Mo and S contents in old leaves was retrieved, other relationships between S and other minerals found under hydroponic conditions, were partly (S and N) or totally hidden (S and Ca) due to multiple interactions occurring under field conditions.

### Test of genericity of the [Mo]:[S] ratio as S-deficiency indicator

The [Mo]:[S] ratio was calculated in leaves of other cultivated species: *B*. *oleracea*, *T*. *aestivum*, *Z*. *mays*, *P*. *sativum* and *S*. *lycopersicum*, grown in controlled conditions. In all species, S content was significantly reduced and Mo content was significantly increased by S deficiency (Table 3). For example, in *B*. *napus* the Mo content increased as early as 1 day after deprivation, in *T*. *aestivum* as early as 2 days and in *Z*. *mays* as early as 5 days of S deficiency, whereas the S content was reduced later. Consequently, the [Mo]:[S] ratio was highly and significantly increased by S deficiency following 1 day of treatment in *B*. *napus*, 2 days in *T*. *aestivum*, 5 days in *Z*. *mays* and this rise was amplified over time. For *B*. *oleracea*, *P*. *sativum* and *S*. *lycopersicum*, no early harvest was performed, but significant increases in leaf Mo content and in the [Mo]:[S] ratio were found after 20, 19 and 65 days, respectively. Values of the [Mo]:[S] ratio were species-dependent, with values ranging from 3 to 250 in control plants and from 27 to 5934 in S-deprived plants, as a result of a high accumulation of Mo in *B*. *napus*, *T*. *aestivum* and in *Z*. *mays*.

**Table 3:** Sulfur content (mg g^-1^ DW) and Mo content (μg g^-1^ DW) and the [Mo]:[S] ratio in leaves of *B*. *napus*, *B*. *oleracea*, *T*. *aestivum*, *Z*. *mays*, *P*. *sativum* and *S*. *lycopersicum* following different S treatments (+S: control treatment, -S: S deficiency treatment) to plants grown under controlled conditions (given in S1 File).

Species	Days after -S treatment	S (mg g^-1^ DW)		Mo (μg g^-1^ DW)		Ratio [Mo]:[S].10^4^	
		+S	-S	+S	-S	+S	-S
***B*.*napus***	0	10.33 ± 0.46		154.6 ± 10.16		149.7 ± 7.34	
	1	10.3 ± 0.39	9.7 ± 0.56	175.3 ± 6.87	237.0 ± 7.70 ***	170.2 ± 1.12	247.4 ± 18.67 **
	21	15.2 ± 0.39	2.3 ± 0.14	308.5 ± 14.02	617.8 ± 40.08 ***	202.2 ± 6.52	2660.4 ± 91.28 ***
***B*. *oleracea***	20	4.8 ± 0.46	1.9 ± 0.04 ***	4.0 ± 0.21	5.0 ± 0.16 **	8.4 ± 0.49	26.5 ± 1.16 ***
***T*. *aestivum***	2	3.6 ± 0.18	3.4 ± 0.22	90.2 ± 9.43	201.5 ± 7.72 ***	249.5 ± 18.17	584.4 ± 24.95 ***
	8	3.5 ± 0.06	3.1 ± 0.00 **	85.1 ± 1.23	719.9 ± 36.62 ***	245.3 ± 5.56	2306.2 ± 110.52 ***
	24	3.4 ± 0.02	1.8 ± 0.05 ***	79.8 ± 2.08	977.1 ± 32.63 ***	236.7 ± 5.58	5285.3 ± 236.13 ***
***Z*. *mays***	0	2.82 ± 0.10		22.6 ± 0.85		80.2 ± 1.54	
	5	3.0 ± 0.03	2.1 ± 0.13 ***	24.7 ± 0.48	468.2 ± 28.15 ***	80.9 ± 0.82	2224.1 ± 72.16 ***
	18	2.4 ± 0.05	0.9 ± 0.04 ***	27.9 ± 1.65	562.6 ± 34.46 ***	116.7 ± 5.06	5934.4 ± 137.28 ***
***P*. *sativum***	0	2.75 ± 0.09		20.5 ± 4.22		73.8 ± 12.95	
	19	2.7 ± 0.10	2.2 ± 0.14 *	9.4 ± 0.50	12.5 ± 0.34 **	34.5 ± 0.67	57.6 ± 5.58 **
***S*. *lycopersicum***	65	10.1 ± 0.71	1.8 ± 0.27 ***	2.9 ± 0.03	5.1 ± 0.82 *	2.9 ± 0.21	27.9 ± 1.96 ***

Data are given as the mean ± SE (n = 4 or n = 3 for *P*. *sativum* data).

*, ** and *** indicate significant differences between control and S-deprived plants for *P*<0.05, *P*<0.01 and P<0.001, respectively.

## Discussion

### Perturbations of Mo homeostasis by non-specific transport of molybdate and sulfate

It is usually assumed that plant nutrient contents are tightly regulated through balanced activities of membrane transporters that mediate the uptake and distribution of nutrients so that a relative compositional homeostasis is maintained. However, under deficiencies, crosstalk induces unavoidable accumulations of some nutrients that upsets the balance and modifies the ionomic composition of plant tissues [23]. In our study, among all mineral nutrients, only Mo uptake was strongly increased by S deficiency in *B*. *napus* (Fig 2) leading to its accumulation in leaves (Fig 3B). From the first day of S deprivation, the Mo content was dramatically increased by 128% in S-deprived plants relative to plants continuously supplied with S. This accumulation of Mo in response of S deficiency has previously been reported in *Brassicaceae* species. Schiavon *et al*. [7] reported that S deficient *B*. *juncea* accumulated significantly more Mo than control plants. Conversely, the supply of S significantly decreased the uptake of Mo by *B*. *oleracea* [29], *B*. *juncea* [28] and by *B*. *napus* [27].

Two root membrane molybdate transporters have been reported so far as responsible for Mo uptake in plants: MOT1 [22] and ASY [26]. In our study, under S deprivation, the expression of *BnaMot1* and *BnaAsy* remained similar to control plants (Fig 3D) and consequently, it may be assumed that the increase in Mo uptake was not a result of an induction of these specific Mo transporters. This is consistent with previous work reporting that root *TaeMot1* expression in *T*. *aestivum* was not significantly affected by S deficiency despite increased concentration of Mo in plant tissues [6]. Indeed, the involvement of MOT1 in plant Mo uptake remains unclear as although *Arabidopsis mot1* mutants and accessions with lower expression of *MOT1* accumulate less Mo, recent results established the localization of MOT1 in mitochondria [23] and not in plasma membranes and in vesicle membranes as previously proposed [22]. Alternatively, some authors have suggested that the accumulation of Mo could be due to non-specific transport by sulfate carriers [7]. Indeed, molybdate and sulfate through their similar chemical properties (charge, size, structure, bonding-properties) could compete for the same binding sites [9, 10]. In our results, others genes (*BnaSutr1*.*1* and *BnaSultr 1*.*2*) encoding high-affinity sulfate transporters of group 1 have been studied in order to clarify their ability to also transport molybdate. The increased uptake of Mo in S-deprived plants was strongly correlated with an increase in *BnaSultr1*.*1* expression at least during the first 7 days (Fig 3D). Similarly, in *T*. *aestivum*, under S deficiency expression of genes encoding the sulfate transporters, *TaeSultr1*.*1* and *TaeSultr4*.*1*, increased and coincided with a 3.7 fold increase in Mo contents in grain [6]. Considering the relationships between Mo uptake and the expression of *BnaSultr1*.*1* (Fig 3D), single mutants of *Arabidopsis thaliana* were used to determine the role of *AtSultr1*.*1* and *AtSultr1*.*2* in the stimulated uptake of Mo during S deficiency (Table 2). Only in the *sultr1*.*2* mutant, the Mo accumulation was reduced compared to Col-0 wildtype under both control and S deficiency conditions, suggesting that this transporter is partly involved in root Mo uptake while increased transcription of *At Sultr1*.*1* could be additionally involved in the leaf accumulation of Mo. However, the *sultr1*.*1* mutant retained the capacity for increased Mo uptake under S deficiency, without increased expression of *AtSultr1*.*2* gene (Table 2). This suggests that others root transporters, responding to S deficiency, may be involved or that activity of root sulfate transporters is also strongly regulated at the post-transcriptional level. Data on a double KO mutant for *atsultr1;1* and *atsultr1;2* can be found on the Purdue ionomics information management system (See [42] and http://www.ionomicshub.org/arabidopsis/piims/showIndex.action) as well as [43]. In this mutant, S and Mo contents are reduced compared to the wild type, suggesting that these two transporters could be involved in the uptake of S and Mo. Therefore, the overall results suggest that a very early increase in Mo uptake following S deprivation is, at least partly, a consequence of the strong and early increased expression of *Sultr1*.*1* and *AtSultr1*.*2* encoding root sulfate transporters.

### A potential indicator derived from early molecular events

Under field conditions, the Mo and S contents also changed in opposite ways in leaves of plants submitted to different S fertilization rates. The lowest Mo contents were found in leaves of plants with the highest S fertilization that had the highest S content. Reciprocally, the highest Mo and lowest S contents were found in leaves of plants supplied with the lowest S fertilizations (Fig 5A–5F and S2 Fig). Similar low Mo content in leaves in S fertilized plants and a range of leaf concentrations (*i*.*e*. between 1 and 3 μg g^-1^ DW) have been previously reported in oilseed rape plants [27] and in cabbage [29] under field conditions. However, the leaf Mo contents obtained in our field experiment were lower than those found under hydroponic conditions. This can easily be explained by the fact that the Mo concentration in the nutrient solution was far higher than in the soil solution, nevertheless, the trend of Mo accumulation during S deficiency was similar. Because of the opposing variations in leaf S and Mo contents, the [Mo]:[S] ratio was lower in plants with an optimal S fertilization than in plants with reduced S fertilization (Fig 5G–5I and S2 Fig). In our study, the [Mo]:[S] ratio therefore discriminated different rates of S fertilization and revealed the S status of plants.

It was also found that the increase in plant growth rates that were triggered by N fertilization increased plant S requirements, resulting in a decrease in the S content in oilseed rape leaves under field conditions (Fig 5A–5C and S2 Fig) as previously reported [44]. A second N fertilization (60 kg ha^-1^) had no major impact on the Mo content of leaves, while the leaf Mo content was lower in N unfertilized plants (Fig 5E compared to Fig 5F and S2 Fig). This lower Mo accumulation found in unfertilized plants could be explained by a reduced growth rate and thus by lower S requirements. As a consequence, N fertilization could interfere in the evolution of the [Mo]:[S] ratio. Indeed, a supplementary N fertilization increased the [Mo]:[S] ratio and the total lack of N fertilization decreased the [Mo]:[S] ratio (Fig 5H and 5I and S2 Fig). Nevertheless, this ratio still allowed differentiation of the plots with different rates of S fertilization.

To allow the effective use of the [Mo]:[S] ratio under field conditions, threshold values are required to estimate the S status of oilseed rape crops, and a first estimation has been calculated from a field experiment using different levels of S fertilization. According to these threshold values, three S status groups were defined: S deficient, at risk of S deficiency and S sufficient, with the additional intermediate class taking into account the area of uncertainty. The study of 45 commercial oilseed rape crops in France suggested that more than 50% were potentially S deficient (Fig 6), highlighting the need for optimizing S fertilization practices. However, more accurate determination of [Mo]:[S] ratio threshold values would require a larger set of experiments during which S fertilization would need to be modulated and the effects on seed yield and quality monitored.

Because oilseed rape is well known for its high S requirements, other cultivated species such as *B*. *oleracea*, *T*. *aestivum*, *Z*. *mays*, *P*. *sativum* and *S*. *lycopersicum* have been tested (Table 3) for their response to S deficiency. In all of them, the leaf [Mo]:[S] ratio was significantly increased by reduced S availability, as a result of a decrease in leaf S content coupled with an accumulation of Mo. The same was true for all three *Arabidopsis* genotypes (Table 2). The general response pattern can also be supported by results from the literature with the same or different species, for example, globe amaranth (*Gomphrena globosa*) [45], cabbage [29], wheat [6] and tomato [11]. A decrease in the [Mo]:[S] ratio resulting from an inadequate S supply seems to be a general response of higher plants, whatever their intrinsic requirements for S.

Overall, the analysis of the leaf [Mo]:[S] ratio is a practical alternative to detect early S deficiency under field conditions, given that molecular indicators require laboratory analysis and use of control plants, which are not easily performed under field conditions. Alternatively, new breakthrough technologies using X ray fluorescence or laser metal analysis, also available as portable field tools, would allow an easy quantification of the [Mo]:[S] ratio.

## Supporting Information

S1 Fig Location of 45 commercial crops in France. Location of 45 commercial crops in Francewith crops classified into three S status groups: S deficient in red, at risk of S deficiency in orange and S sufficient in green. Map of France from Institut Géographique National (IGN, 2016, free of copyrights).
(TIF)

S2 Fig S and Mo contents and [Mo]:[S] ratio in young leaves grown under field conditions. (A, B, C) S content (mg g^-1^ DW), (D, E, F) Mo content (μg g^-1^ DW) and (G, H, I) the [Mo]:[S] 10^4^ ratio (to simplify reading the [Mo]:[S] ratio is presented with a multiplier factor of 10^4^) in young leaves of *B*. *napus* grown under field conditions after (A, D, G) 15 and (B, C, E, F, H, I) 47 days of fertilization. Plants received no mineral fertilization (hatched bars, 0 kg S.ha^-1^, 0 kg N.ha^-1^), or 0 kg S ha^-1^ (white bar), 12 kg S ha^-1^ (grey bar) or 36 kg S ha^-1^ (black bar) with 65 kg N ha^-1^ (dashed bars) or 125 kg N ha^-1^ (full bars). Within the same graph, letters when different between fertilization treatments indicate significant differences for *P*<0.05.
(TIF)

S3 Fig Principal component analysis of mineral contents (N, P, K, S, Ca, Mg, Fe, Cu, Zn, Mn, Mo, B and Na) in old leaves of 45 commercial oilseed rape crops. Correlation circles on the factorial planes (A) and (B) projection of the commercial crops (numbers refer to fields/crops given in Fig 6 and SD2). S deficient in red, at risk of S deficiency in orange and S sufficient in green.
(TIF)

S1 File Growth conditions used for multispecies experiment. (DOCX)

S1 Table Composition of the two nutrient solutions used for control and S deprivation during the treatment period of *B*. *napus and Z*. *mays*. Nutrient concentrations are expressed in mM.
(PPTX)

## References

[1] Nacry P, Bouguyon E, Gojon A. Nitrogen acquisition by roots: physiological and developmental mechanisms ensuring plant adaptation to a fluctuating resource. Plant Soil. 2013; 370: 1–29.

[2] Ashley MK, Grant M, Grabov A. Plant responses to potassium deficiencies: a role for potassium transport proteins. J Exp Bot. 2006; 57: 425–436. 10.1093/jxb/erj034 16364949

[3] Hawkesford MJ. Transporter gene families in plants: the sulphate transporter gene family—redundancy or specialization? Physiol Plant. 2003; 117: 155–163.

[4] Malvi UR. Interaction of micronutrients with major nutrients with special reference to potassium. J Agric Sci. 2011; 24: 106–109.

[5] Schachtman D, Liu W. Molecular pieces to the puzzle of the interaction between potassium and sodium uptake in plants. Trends Plant Sci. 1999; 4: 281–287. 10407444 10.1016/s1360-1385(99)01428-4

[6] Shinmachi F, Buchner P, Stroud JL, Parmar S, Zhao F-J, McGrath SP et al Influence of sulfur deficiency on the expression of specific sulfate transporters and the distribution of sulfur, selenium, and molybdenum in wheat. Plant Physiol. 2010; 153: 327–336. 10.1104/pp.110.153759 20219830 PMC2862427

[7] Schiavon M, Pittarello M, Pilon-Smits EAH, Wirtz M, Hell R, Malagoli M. Selenate and molybdate alter sulfate transport and assimilation in *Brassica juncea* L. Czern.: Implications for phytoremediation. Environ Exp Bot. 2012; 75: 41–51.

[8] Mendel RR. Molybdenum: biological activity and metabolism. Dalton Trans Camb Engl. 2005; 2003: 3404–3409. 10.1039/b505527j 16234918

[9] Dudev T, Lim C. Oxyanion selectivity in sulfate and molybdate transport proteins: An ab Initio/CDM Study. J Am Chem Soc. 2004; 126: 10296–10305. 10.1021/ja047951n 15315443

[10] Bittner F (2014) Molybdenum metabolism in plants and crosstalk to iron. Front Plant Sci. 5: 28 10.3389/fpls.2014.00028 24570679 PMC3916724

[11] Alhendawi RA, Kirkby EA, Pilbeam DJ. Evidence that sulfur deficiency enhances molybdenum transport in xylem sap of tomato plants. J Plant Nutr. 2005; 28: 1347–1353.

[12] Kopriva S, Mugford SG, Matthewman C, Koprivova A. Plant sulfate assimilation genes: redundancy versus specialization. Plant Cell Rep. 2009; 28: 1769–1780. 10.1007/s00299-009-0793-0 19876632

[13] Cao MJ, Wang Z, Wirtz M, Hell R, Oliver DJ, Xiang CB. SULTR3;1 is a chloroplast-localized sulfate transporter in *Arabidopsis thaliana*. Plant J. 2013; 73: 607–616. 10.1111/tpj.12059 23095126

[14] Kataoka T, Watanabe-Takahashi A, Hayashi N, Ohnishi M, Buchner P et al Vacuolar sulfate transporters are essential determinants controlling internal distribution of sulfate in *Arabidopsis*. Plant Cell. 2004; 16: 10, 2693–2704. 10.1105/tpc.104.023960 15367713 PMC520965

[15] Llamas A, Tejada-Jiménez M, Fernández E, Galván A (2011) Molybdenum metabolism in the alga Chlamydomonas stands at the crossroad of those in Arabidopsis and humans. Met Integr Biometal Sci 3: 578–590. 10.1039/c1mt00032b 21623427

[16] Takahashi H, Watanabe-Takahashi A, Smith FW, Blake-Kalff M, Hawkesford MJ, Saito K. The roles of three functional sulphate transporters involved in uptake and translocation of sulphate in *Arabidopsis thaliana*. The Plant J. 2000; 23: 171–182. 10929111 10.1046/j.1365-313x.2000.00768.x

[17] Abdallah M, Dubousset L, Meuriot F, Etienne P, Avice J-C, Ourry A. Effect of mineral sulphur availability on nitrogen and sulphur uptake and remobilization during the vegetative growth of *Brassica napus* L. J Exp Bot. 2010; 61: 2635–2646. 10.1093/jxb/erq096 20403880 PMC2882259

[18] Parmar S, Buchner P, Hawkesford MJ. Leaf developmental stage affects sulfate depletion and specific sulfate transporter expression during sulfur deprivation in *Brassica napus* L. Plant Biol Stuttg Ger. 2007; 9: 647–653. 10.1055/s-2007-965428 17853364

[19] Casieri L, Gallardo K, Wipf D. Transcriptional response of *Medicago truncatula* sulphate transporters to arbuscular mycorrhizal symbiosis with and without sulphur stress. Planta. 2012; 235: 1431–1447. 10.1007/s00425-012-1645-7 22535379

[20] Fitzpatrick KL, Tyerman SD, Kaiser BN. Molybdate transport through the plant sulfate transporter SHST1. FEBS Lett. 2008; 582: 1508–1513. 10.1016/j.febslet.2008.03.045 18396170

[21] Tejada-Jiménez M, Llamas Á, Sanz-Luque E, Galván A, Fernández E. A high-affinity molybdate transporter in eukaryotes. Proc Natl Acad Sci. 2007; 104: 20126–20130. 10.1073/pnas.0704646104 18077439 PMC2148433

[22] Tomatsu H, Takano J, Takahashi H, Watanabe-Takahashi A, Shibagaki N, Fujiwara T. An *Arabidopsis thaliana* high-affinity molybdate transporter required for efficient uptake of molybdate from soil. Proc Natl Acad Sci. 2007; 104: 18807–18812. 10.1073/pnas.0706373104 18003916 PMC2141858

[23] Baxter I, Muthukumar B, Park HC, Buchner P, Lahner B, Danku J et al Variation in molybdenum content across broadly distributed populations of *Arabidopsis thaliana* is controlled by a mitochondrial molybdenum transporter (MOT1). PLoS Genet. 2008; 4: e1000004 10.1371/journal.pgen.1000004 18454190 PMC2265440

[24] Gasber A, Klaumann S, Trentmann O, Trampczynska A, Clemens S, Schneider S et al Identification of an Arabidopsis solute carrier critical for intracellular transport and inter-organ allocation of molybdate. Plant Biol. 2011; 13: 710–718. 10.1111/j.1438-8677.2011.00448.x 21815974

[25] Tejada-Jiménez M, Galván A, Fernández E. Algae and humans share a molybdate transporter. Proc Natl Acad Sci. 2011; 108: 6420–6425. 10.1073/pnas.1100700108 21464289 PMC3080982

[26] Hibara K-I, Hosoki W, Hakoyama T, Ohmori Y, Fujiwara T, Itoh J-I et al Abnormal Shoot in Youth, a homolog of molybdate transporter gene, regulates early shoot development in rice. Am J Plant Sci. 2013; 4: 1–9.

[27] Balík J, Pavlíková D, Tlustos P, Sykora K, Cerny J. The fluctuation of molybdenum content in oilseed rape plants after the application of nitrogen and sulphur fertilizers. Plant Soil Environ. 2006; 52: 301–307.

[28] Harris J, Schneberg KA, Pilon-Smits EAH. Sulfur-selenium-molybdenum interactions distinguish selenium hyperaccumulator *Stanleya pinnata* from non-hyperaccumulator *Brassica juncea* (Brassicaceae). Planta. 2014; 239: 479–491. 10.1007/s00425-013-1996-8 24233101

[29] Hunashikatti MG, Channal HT, Sarangamath PA, Manjunathaiah HM, Hebsur NS. Effect of sulphur and molybdenum on the dry matter yield and uptake of S and Mo by cabbage. Karnataka J Agric Sci. 2000; 13:840–845.

[30] Janzen HH, Bettany JR. Sulfur nutrition of rapeseed: I. Influence of fertilizer nitrogen and sulfur rates. Soil Sci Soc Am J. 1984; 48: 100–107.

[31] McGrath SP, Zhao FJ. Sulphur uptake, yield responses and the interactions between nitrogen and sulphur in winter oilseed rape (*Brassica napus*). J Agric Sci. 1996; 126: 53–62.

[32] Scherer HW. Sulphur in crop production—invited paper. Eur J Agron. 2001; 14: 81–111.

[33] Malhi SS, Gan Y, Raney JP. Yield, seed quality, and sulfur uptake of oilseed crops in response to sulfur fertilization. Agron J. 2007; 99: 570–577.

[34] D’Hooghe P, Dubousset L, Gallardo K, Kopriva S, Avice J-C, Trouverie J. Evidence for proteomic and metabolic adaptations Associated with alterations of seed yield and quality in sulfur-limited *Brassica napus* L. Mol Cell Proteomics. 2014; 13: 1165–1183. 10.1074/mcp.M113.034215 24554741 PMC4014277

[35] Blake-Kalff MMA, Zhao F-J, Hawkesford MJ, McGrath SP. Using plant analysis to predict yield losses caused by sulphur deficiency. Ann Appl Biol. 2001; 138: 123–127.

[36] Sorin E, Etienne P, Maillard A, Zamarreno AM, Garcia-Mina JM, Arkoun M et al Effect of sulphur deprivation on osmotic potential components and nitrogen metabolism in oilseed rape leaves: identification of a new early indicator. J Exp Bot. 2015; 66, 20:6175–6189. 10.1093/jxb/erv321 26139826

[37] Blake-Kalff MMA, Hawkesford MJ, Zhao FJ, McGrath SP. Diagnosing sulfur deficiency in field-grown oilseed rape (Brassica napus L.) and wheat (Triticum aestivum L.). Plant Soil. 2000; 225: 95–107.

[38] Shibagaki N, Rose A, McDermott JP, Fujiwara T, Hayashi H, Yoneyama T et al Selenate-resistant mutants of *Arabidopsis thaliana* identify Sultr1;2, a sulfate transporter required for efficient transport of sulfate into roots. Plant J. 2002; 29: 475–486. 11846880 10.1046/j.0960-7412.2001.01232.x

[39] Sarda X, Diquelou S, Abdallah M, Nesi N, Cantat O, Le Gouee P et al Assessment of sulphur deficiency in commercial oilseed rape crops from plant analysis. J Agr Sci. 2014; 152: 616–633

[40] Livak KJ, Schmittgen TD (2001) Analysis of relative gene expression data using real-time quantitative PCR and the 2(T)(-Delta Delta C) method. Methods 25: 402–408. 10.1006/meth.2001.1262 11846609

[41] Koprivova A, Giovannetti M, Baraniecka P, Lee BR, Grondin C, Loudet O, et al Natural Variation in the ATPS1 isoform of ATP sulfurylase contributes to the control of sulfate levels in *Arabidopsis*. Plant Physiol. 2013; 163, 3: 1133–1141. 10.1104/pp.113.225748 24027241 PMC3813639

[42] Baxter I, Ouzzani M, Orcun S, Kennedy B, Jandhyala SS, Salt DE. Purdue ionomics information management system. An integrated functional genomics platform. Plant Physiol. 2007; 143: 600–611. 10.1104/pp.106.092528 17189337 PMC1803751

[43] Huang X-Y, Chao D-Y, Koprivova A, Danku J, Wirtz M, Müller S et al Nuclear localised MORE SULPHUR ACCUMULATION1 epigenetically regulates sulphur homeostasis in *Arabidopsis thaliana*. PLoS Genet. 2016; 12(9): e1006298 10.1371/journal.pgen.1006298 27622452 PMC5021336

[44] Zhao F, Evans EJ, Bilsborrow PE, Syers JK. Influence of sulphur and nitrogen on seed yield and quality of low glucosinolate oilseed rape (*Brassica napus* L). J Sci Food Agric. 1993; 63: 29–37.

[45] Wang MY, Wu LH, Zhang J. Impacts of root sulfate deprivation on growth and elements concentration of globe amaranth (Gomphrena globosa L.) under hydroponic condition. Plant Soil Env. 2009; 55, 11: 484–493.

