# Supplementary figures and images for: Non-Specific Root Transport of Nutrient Gives Access to an Early Nutritional Indicator: The Case of Sulfate and Molybdate
Source: PLoS One. 2016 Nov 21;11(11):e0166910. doi: 10.1371/journal.pone.0166910 (PMC5117742; doi:10.1371/journal.pone.0166910)

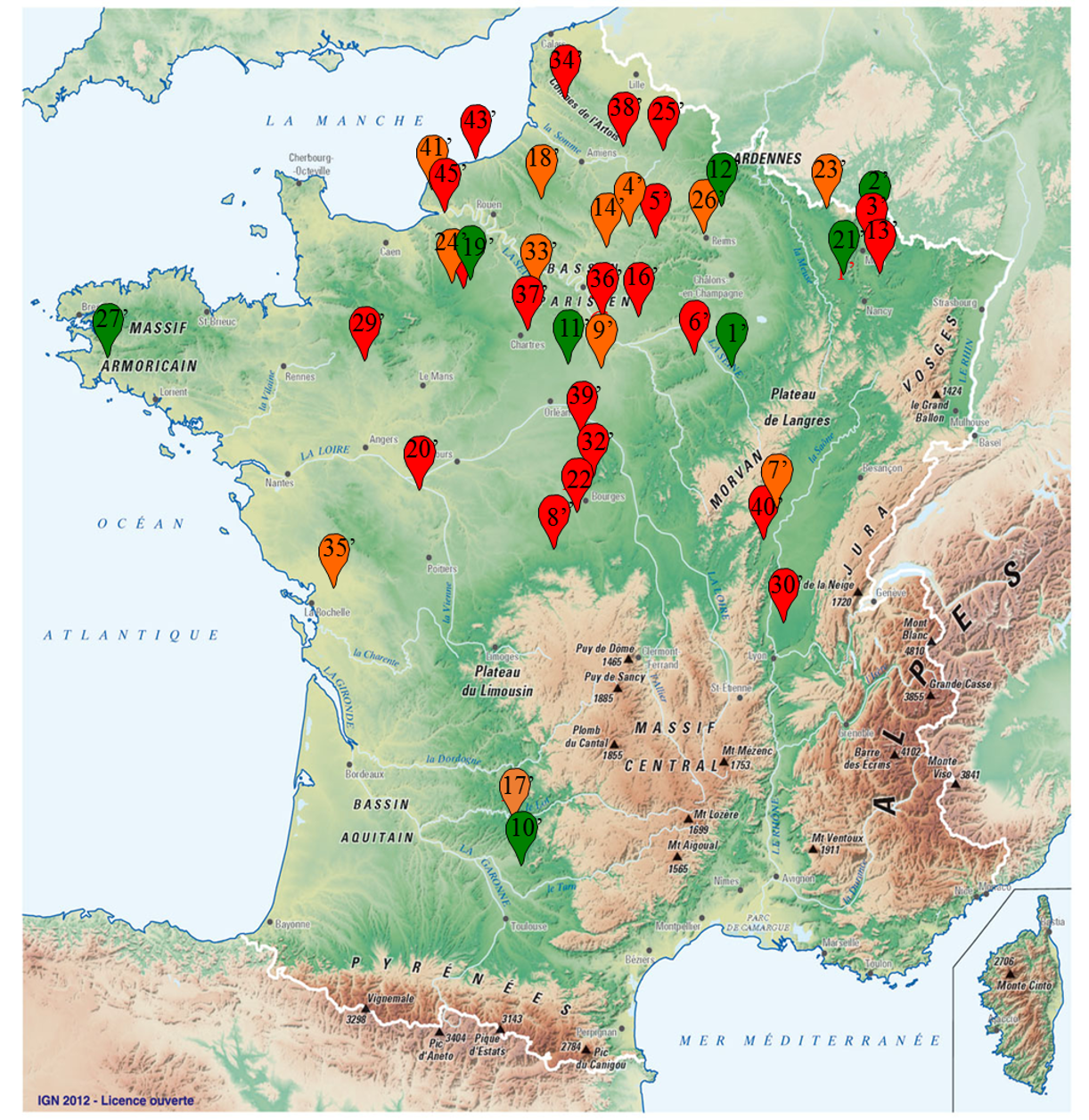

Supplement: S1 Fig — Location of 45 commercial crops in Francewith crops classified into three S status groups: S deficient in red, at risk of S deficiency in orange and S sufficient in green. Map of France from Institut Géographique National (IGN, 2016, free of copyrights). (TIF) [file pone.0166910.s001.tif]

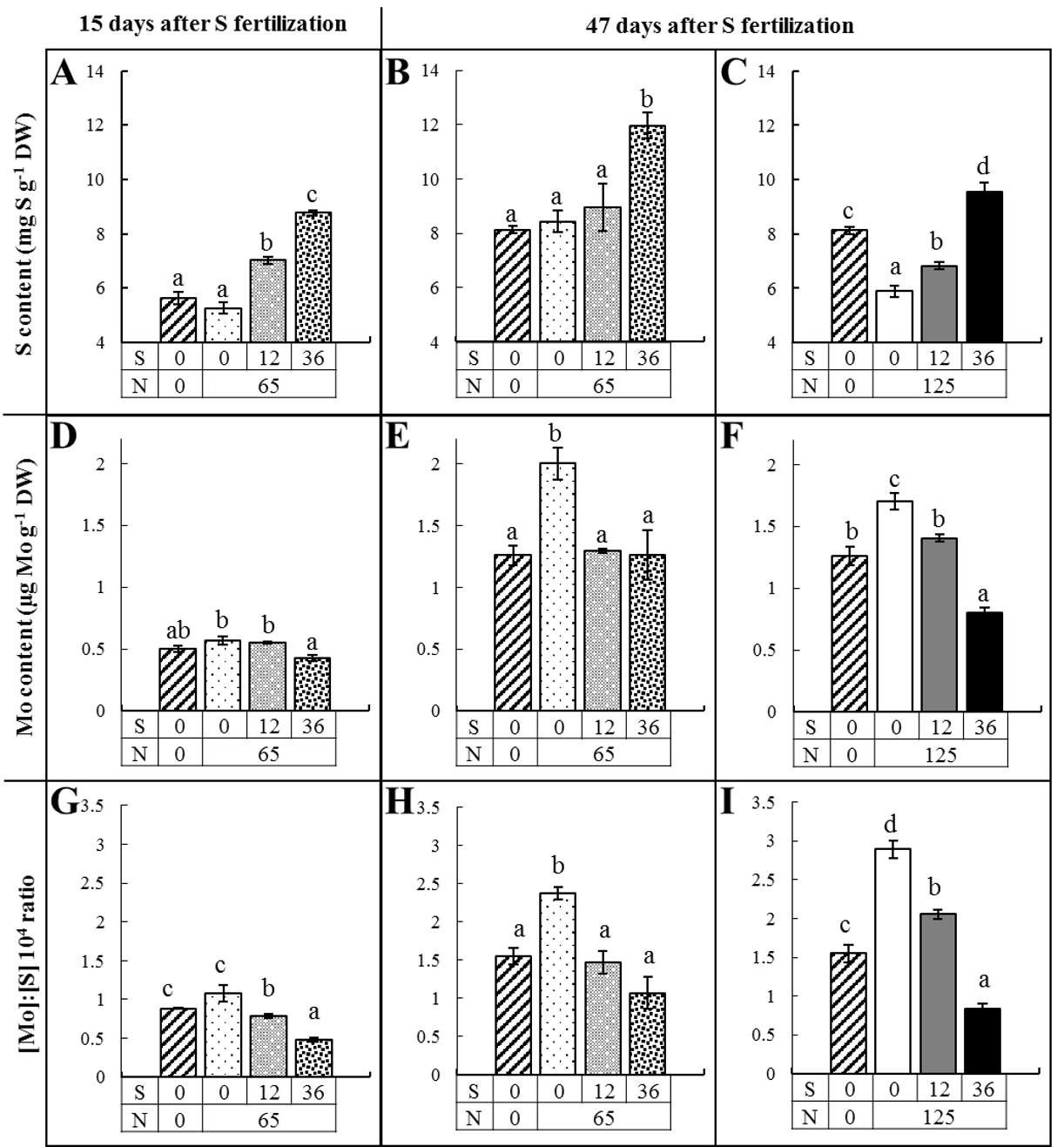

Supplement: S2 Fig — (A, B, C) S content (mg g-1 DW), (D, E, F) Mo content (μg g-1 DW) and (G, H, I) the [Mo]:[S] 104 ratio (to simplify reading the [Mo]:[S] ratio is presented with a multiplier factor of 104) in young leaves of B. napus grown under field conditions after (A, D, G) 15 and (B, C, E, F, H, I) 47 days of fertilization. Plants received no mineral fertilization (hatched bars, 0 kg S.ha-1, 0 kg N.ha-1), or 0 kg S ha-1 (white bar), 12 kg S ha-1 (grey bar) or 36 kg S ha-1 (black bar) with 65 kg N ha-1 (dashed bars) or 125 kg N ha-1 (full bars). Within the same graph, letters when different between fertilization treatments indicate significant differences for P<0.05. (TIF) [file pone.0166910.s002.tif]

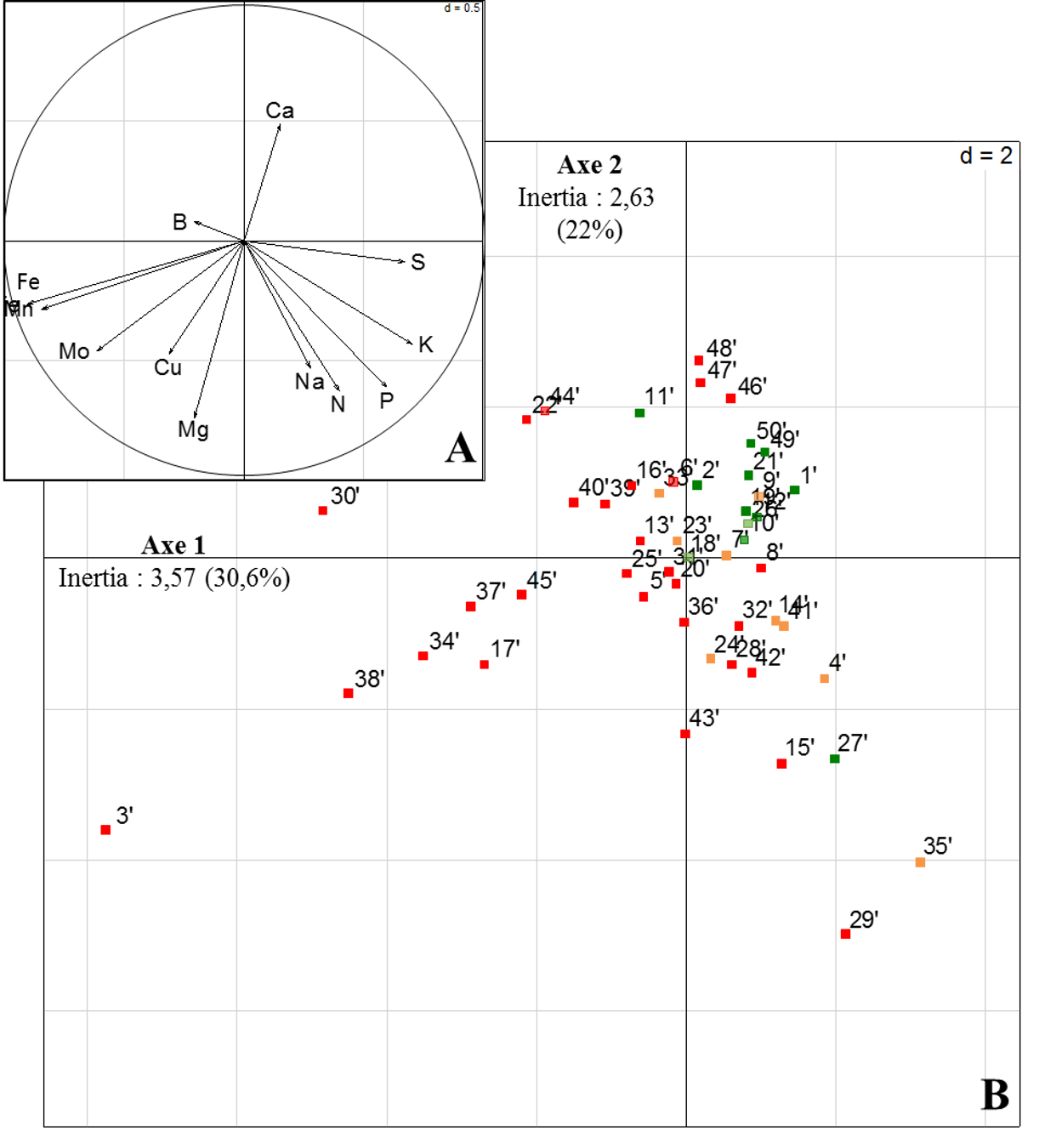

Supplement: S3 Fig — Correlation circles on the factorial planes (A) and (B) projection of the commercial crops (numbers refer to fields/crops given in Fig 6 and SD2). S deficient in red, at risk of S deficiency in orange and S sufficient in green. (TIF) [file pone.0166910.s003.tif]
