# Supplementary material for: Non-Specific Root Transport of Nutrient Gives Access to an Early Nutritional Indicator: The Case of Sulfate and Molybdate
Source: PLoS One. 2016 Nov 21;11(11):e0166910. doi: 10.1371/journal.pone.0166910 (PMC5117742; doi:10.1371/journal.pone.0166910)
Supplement: S1 File — (DOCX) [file pone.0166910.s004.docx]

*Z. mays*

Seeds of *Z. mays* cv Ronaldinio were germinated on perlite over demineralized water for seven days in the dark and then five days under natural light. Just after first leaf emergence, seedlings were transferred to hydroponic conditions (18 seedlings per 20L-plastic tank) in a greenhouse, between March and April, with a thermoperiod of 20°C (day) and 15°C (night). Natural light was supplemented with high-pressure sodium lamps (Master Greenpower T400W, Philips, Amsterdam, Netherlands) (350 µmol m^-2^ s^-1^ of photosynthetically active radiation at the canopy height) for 16h. The aerated nutrient solution contained: 3.75 mM KNO_3_, 0.5 mM MgSO_4_, 0.5 mM CaCl_2_, 0.25 mM KH_2_PO_4_, 0.2 mM EDTA‑2NaFe, 14 µM H_3_BO_3_, 5 µM MnSO_4_, 3 µM ZnSO_4_, 0.7 µM CuSO_4_, 0.7 µM (NH_4_)_6_Mo_7_O_24_, 0.1 µM CoCl_2_, 0.04 µM NiCl_2_ and was buffered to pH 6.6 with 0.91 mM CaCO_3_. This solution was renewed according to the rate of NO_3_^-^ depletion monitored daily by using NO_3_^-^ test strips (Merck Millipore, Darmstadt, Germany) in order to maintain optimal nutrition conditions. After 8 days of growth, plants were separated into two batches supplied with a modified nutrient solution chosen in order to achieve S deficiency and to maintain the same concentration of other nutrients (Supplemental data SD1): (i) control plants (control) were grown with 508.7 µM SO_4_^2-^, (ii) S limited plants (-S) were grown with 8.7 µM SO_4_^2^. Nutrient solutions were also renewed according to NO_3_^-^ depletion by monitoring the NO_3_^-^ level in the tank.

*T. aestivum*

Seeds of *T. aestivum* L. cv Sankara were germinated on perlite over demineralized water for five days in the dark and then five days under light. Seedlings were transferred to hydroponic conditions (30 seedlings per 7L-plastic tank) in a growth chamber, with a thermoperiod of 22°C (day) and 18°C (night). Plants received artificial light provided by neon lamps (Lumilux cool daylight, 36W, Osram, Munich, Germany) (100 µmol m^-2^ s^-1^ of photosynthetically active radiation at the canopy height) for 12h. The aerated nutrient solution contained: 2 mM KNO_3_, 1 Ca(NO_3_)_2_, 0.5 mM MgSO_4_, 1 mM KH_2_PO_4_, 0.1 mM EDTA‑2NaFe, 23 µM H_3_BO_3_, 5 µM MnSO_4_, 2 µM ZnSO_4_, 0.9 µM CuSO_4_, 0.3 µM (NH_4_)_6_Mo_7_O_24_, 0.1 µM CoCl_2_ and was buffered to pH 6 with KOH. This solution was renewed every two or three days. After 11 days of growth, plants were separated into two batches: (i) control plants were grown with 507.9 µM SO_4_^2-^, (ii) S limited plants were grown with 7.9 µM SO_4_^2-^, replacing MgSO_4_ by MgCl_2_.

*B. oleracea*

*B. oleracea* cv Nikolas plants were grown individually in pots filled with a mixture of perlite: vermiculite (v:v, 1:1) in 1L pots for one month then in 2L pots. Plants were grown in a growth chamber, with temperatures of 21°C during the day and 18°C during the night, a 14h photoperiod and a mean photosynthetically active radiation of 100 µmol m^-2^ s^-1^ was guaranteed by the use of neon lamps (Lumilux soft white, L58W/840, Osram, Munich, Germany). Plants were watered throughout the experiment, every two days with a nutrient solution composed of 4.5 mM KNO_3_, 3.6 Ca(NO_3_)_2_, 1.4 NH_4_NO_3_, 0.3 mM MgSO_4_, 0.1 mM MgCl_2_, 1 mM KH_2_PO_4_, 35 mg l^-1^ Fe-EDTA (FerVeg E13, Angibaud et spécialité, La Rochelle, France) and 3.5 mg l^-1^ OligoMix (Oligoveg S2, Angibaud et spécialité, La Rochelle, France). For S deficiency treatment the same solution described above was used except that MgSO_4_ was removed and MgCl_2_ was adjusted to 0.5 mM.

*S. lycopersicum*

*S. lycopersicum* cv Plaisance on Emperador was grown in a greenhouse between April and July under natural light temperatures controlled to 20°C during the day and 15°C during the night. One plant per 1L pot was cultivated for one month and transferred to 3L pots filled with a mixture of perlite: vermiculite (v:v, 1:1) and were watered every day by a nutrient solution containing: 5.2 mM KNO_3_, 6.2 Ca(NO_3_)_2_, 0.5 NH_4_NO_3_, 1.8 mM KH_2_PO_4_, 1.9 mM MgSO_4_0.3 mM MgCl_2,_ 15 mg l^-1^ Fe-EDTA (FerVeg E13, Angibaud et spécialité, La Rochelle, France) and 3.5 mg l^-1^ OligoMix (Oligoveg S2, Angibaud et spécialité, La Rochelle, France). For S deficiency treatment the same solution described above was used except that MgSO_4_ was removed and MgCl_2_ was adjusted to 2.2 mM.

*P. sativum*

*P. sativum* L. plants (genotype Cameor) were grown in a randomized complete-block design in a greenhouse temperature controlled to 19°C during the day and 15°C during the night. Artificial lighting was used to reach 16h light per day: 400 W sodium lamps, 6 lamps in 17.32 m^2^, radiation in the range 400-700 nm, and photosynthetic characteristics of 695 µmol s^-1^. Pea seeds were directly sown at 2 cm depth in 2L pots containing 25% sand (type B3, Biot, France) and 75% perlite. Thee mature seeds were sown per pot, then after emergence, only one seedling was kept in each pot. Control plants were supplied with a nutrient solution containing 4 mM KNO_3_, 2 mM Ca(NO_3_)_2_, 0.3 mM MgSO_4_, 0.9 mM MgCl_2_, 0.2 mM NaCl, 0.72 µM Na_2_MoO_4_, 0.10 mM FeNa-EDTA, 8.2 µM MnCl_2_, 1 µM CuCl_2_, 1 µM ZnCl_2_, 30 µM H_3_BO_3_, 1 mM K_2_HPO_4_ (pH adjusted to 6.3 using H_3_PO_4_ before addition of K_2_HPO_4_). An automatic plant watering system, with two drippers per pot, was used for all plants. For applying S deficiency (at the eight-leaf stage), the pots were rinsed with 4x2L of deionized water and SO_4_^2-^ from the solution described above was replaced by 1.16 mM MgCl_2_.
