# Supplementary material for: Non-Specific Root Transport of Nutrient Gives Access to an Early Nutritional Indicator: The Case of Sulfate and Molybdate
Source: PLoS One. 2016 Nov 21;11(11):e0166910. doi: 10.1371/journal.pone.0166910 (PMC5117742; doi:10.1371/journal.pone.0166910)
Supplement: S1 Table — Nutrient concentrations are expressed in mM. (PPTX) [file pone.0166910.s005.pptx]

## Slide 1
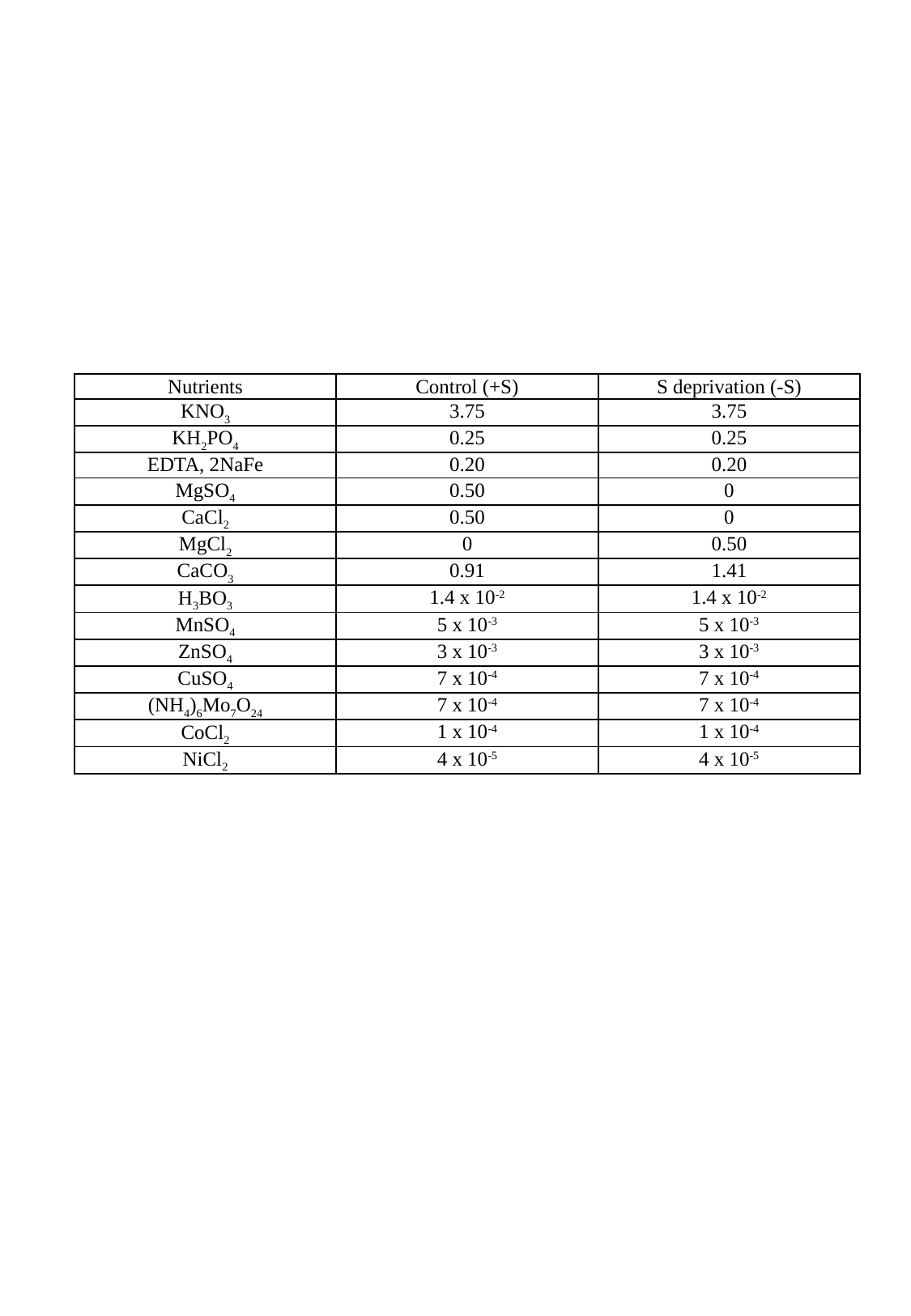

| Nutrients | Control (+S) | S deprivation (-S) |
| --- | --- | --- |
| KNO3 | 3.75 | 3.75 |
| KH2PO4 | 0.25 | 0.25 |
| EDTA, 2NaFe | 0.20 | 0.20 |
| MgSO4 | 0.50 | 0 |
| CaCl2 | 0.50 | 0 |
| MgCl2 | 0 | 0.50 |
| CaCO3 | 0.91 | 1.41 |
| H3BO3 | 1.4 x 10^-2^ | 1.4 x 10^-2^ |
| MnSO4 | 5 x 10^-3^ | 5 x 10^-3^ |
| ZnSO4 | 3 x 10^-3^ | 3 x 10^-3^ |
| CuSO4 | 7 x 10^-4^ | 7 x 10^-4^ |
| (NH4)6Mo7O24 | 7 x 10^-4^ | 7 x 10^-4^ |
| CoCl2 | 1 x 10^-4^ | 1 x 10^-4^ |
| NiCl2 | 4 x 10^-5^ | 4 x 10^-5^ |
